# What Pennsylvanians Are Thankful for

**Published:** 1898-04

**Authors:** 


### WHAT PENNSYLVANIANS ARE THANKFUL FOR.

A PROSPEROUS, advancing, and united State organization.

For several earnest and aggressive local societies.

That our Veterinary Department has now a veterinarian as Dean.

For one of the most harmonious State Livestock Sanitary Boards, and of the excellent work it is doing for every interest concerned.

For the presence within our borders of one of the most enterprising veterinary journals.

For a State Veterinarian whose interest in the welfare of the whole profession is broad and generous.

For a Governor who has many times shown his keen appreciation in the value of veterinary work throughout our State.

For a biological laboratory where veterinarians are largely employed, and where are produced most excellent products for veterinary uses.

For a State Veterinary Examining Board and proper laws for the regulation of the practice of veterinary science.

For a large number of members of the profession whose increasing years add only to their keener interest in veterinary progress.

For the prospect of adding to the world's progress in an experiment building specially designed for the study of infectious and contagious disease of livestock.

For the growth and increased recognition of her veterinarians and their work as such on municipal boards of health, departments of public safety, and by individuals and corporations handling large amounts of dairy products.

For the special interest in our work of many of the health bodies of our State and many members of the medical profession.

For the increased interest in our Veterinary Department by Provost Harrison.

For the increasing interest of the members of our profession in the work of the national organization.

For the promised return of better times among our livestock industries and a greater demand for our services.

For a promised gathering at Pittsburg at our semi-annual meeting in September, the largest in our history.

For officers in all our veterinary organizations whose fondest hopes are to outrival in progress their predecessors.

